# Does Early Exposure to Chinese–English Biliteracy Enhance Cognitive Skills?

**DOI:** 10.3389/fpsyg.2022.852437

**Published:** 2022-04-25

**Authors:** Jing Yin, Connie Qun Guan, Elaine R. Smolen, Esther Geva, Wanjin Meng

**Affiliations:** ^1^School of Languages and Communication Studies, Beijing Jiaotong University, Beijing, China; ^2^Beijing Language and Culture University, Beijing, China; ^3^Department of Psychology, Carnegie Mellon University, Pittsburgh, PA, United States; ^4^Teachers College, Columbia University, New York, NY, United States; ^5^The Ontario Institute for Studies in Education, University of Toronto, Toronto, ON, Canada; ^6^Department of Moral, Psychology and Special Education, China National Institute of Education Sciences, Beijing, China

**Keywords:** early exposure, morphological awareness, word recognition, working memory, attention, Chinese–English bi-scriptal exposure, children

## Abstract

Clarifying the effects of biliteracy on cognitive development is important to understanding the role of cognitive development in L2 learning. A substantial body of research has shed light on the cognitive factors contributing to biliteracy development. Yet, not much is known about the effect of the degree of exposure to biliteracy on cognitive functions. To fill this research void, we measured three categories of biliteracy skills (language, reading, and cognitive–linguistic skills in both Chinese and English) jointly and investigated the effects of biliteracy skill performance in these three categories on cognitive skills (working memory and attentional control) in Chinese L1 children who were exposed to English as L2 beginning at age 3 (“early”) or in grade 3 (“late”). In this cross-sectional study, 10 parallel Chinese and English language, reading, and cognitive–linguistic measures were administered to emerging Chinese–English bilingual children in grade 3 (*n* = 178) and grade 6 (*n* = 176). The results revealed that early exposure to Chinese–English biliteracy enhanced cognitive skills but with a cost of a slight delay in performance on Chinese L1 language skills in grade 3 (but not in grade 6). Importantly, the present findings suggest that, in addition to universal and developmental processes, the cumulative effect of early and sustained bi-scriptal exposure enhances working memory and attention in school children.

## Introduction

Exposure to Chinese–English biliteracy has recently become a common experience for children in China as China has the largest-scale English teaching and learning around the world and has expanded English teaching and learning into kindergartens (Chen, 2015). Managing two languages is a daunting task that may exceed children’s cognitive resources and thus could potentially lead to some intellectual issues (Peal and Lambert, 1962; Bialystok et al., 2012; Abu-Shnein, 2021). From the very beginning, Chinese–English bilingual research targeting children was concerned with the domains of linguistic (Wang et al., 2005; Cheng et al., 2011) and metalinguistic skills (Cheung et al., 2010; Luo et al., 2014), just as it is now. Previous research tended to explore whether and to what extent cognitive factors contribute to biliteracy development among children (Bialystok and Hakuta, 1999). At the same time, more recent research has begun to focus on the extent to which bilingual exposure enhances cognitive factors (Barac et al., 2014). The current study set out to explore whether and to what extent the degree of exposure to Chinese–English biliteracy affects cognitive functions among Chinese children.

We aim to examine the effects of language, reading, and cognitive–linguistic performance on cognitive skills in two groups varying in the extent of exposure to their L2. Both groups of Chinese–English bilinguals were elementary school children, but they differed in the age at which they began to receive systematic exposure to language and literacy in the L2 (i.e., early vs. late). We hypothesized that there would be differences in linguistic and cognitive skills between these two groups, that some linguistic component skills would predict cognitive skills, and that the contribution of the linguistic to cognitive performance would be mediated by degree of exposure cross-sectionally (i.e., grade 3 vs. 6).

### Features of Chinese and English Biliteracy

The paths to acquiring Chinese and English appear to be quite different. English is an alphabetic system in which 26 letters are associated with phonemes, which are combined to form words. In contrast, Chinese is a logographic language whose basic unit is a character, and the graphemes do not map to individual phonemes but rather to syllabic morphemes (Wang et al., 2005). Hence, Chinese–English bilingual children may concurrently develop both general and specific cognitive learning mechanisms and strategies that may be uniquely associated with each written language and, along the way, develop metalinguistic awareness of how the two languages vary from each other, which can in turn enhance further learning.

Chinese is a morphosyllabic writing system comprising more than 50,000 characters, of which about 5,000 are commonly used in everyday life (Gao et al., 2019). Pinyin, a phonetic transcription of Chinese characters, was established by the Chinese government in 1958 (Zhou, 1958) to support the pronunciation of characters. Most scholars refer to the Chinese writing system as logographic or morphographic (e.g., Handel, 2013, 2014), in which the relationship between graphemes and morphemes is the most important factor for language learning (Rogers, 2005). Chinese writing is formed by visually complex characters (Fung et al., 2020). Left–right structure, up–down structure, and isolated characters are typical Chinese character structures. More than 80 percent of words in Chinese consist of two characters that are usually a combination of both semantic radicals and phonetic radicals, providing semantic cues and phonological hints, respectively (Kang, 1993; Taylor and Taylor, 1995). As a paratactic language, Chinese has many homophones that entail different graphic but the same phonetic forms. For instance, 克(kè) (which means *to control*, *to restrain* or represents “*gram*” in English), 课(kè) (which means *class* or *lesson* in English), and 客(kè) (which means *a guest* or *a consumer* in English) are homophones with radically different graphic forms and different meaning. Due to this characteristic of the Chinese language, verbal word recognition requires specific perceptual abilities, and morphological awareness requires language users to pay special attention to grapheme–morpheme relations (Koda, 2016). Practicing these Chinese linguistic skills with such specific script features should thus have an impact on the development of cognitive skills.

English, on the other hand, is an alphabetic language consisting of 26 letters of Latin script, with phonological and morphologic features that are very different from those of Chinese. First, phonological awareness is essential for learning to read and spell in alphabetic languages such as English (Wagner and Torgesen, 1987). English has a different syllabic structure from Chinese (Goswami et al., 2011). For instance, in English, a syllable can be divided into an onset and rime. The onset can contain a consonant (e.g., −d in *dog*) or consonant clusters (e.g., −dr in *drink*). The rime contains a vowel (e.g., −aʊ in *how*) or a vowel plus consonants (e.g., −og in *dog*), or a vowel plus a consonant cluster (e.g., −rp in *sharp*). Phonological forms are thus important for word segmentation and word recognition in English. In Chinese, a syllable can be divided into two sub-syllabic components: the initial part (*shengmu*, i.e., the initial consonant of a Chinese syllable) and the final component (*yunmu*, i.e., the simple or compound vowel of a Chinese syllable; Třísková, 2011).

Second, to better acquire a language, it is necessary to understand the meaning and grammatical role of different morphemes and knowledge of the morphemic structure of words (Carlisle and Feldman, 1995). In a language such as English, morphemes include inflections (help/helped; see/sees/saw), derivations (help/helpful), and compounds (foot + ball = football; help + line = helpline). In English, derivational words are extremely common, while inflections are less so (Chen and Schwartz, 2018). While inflections and derivations do not exist in Chinese, most Chinese words are compounds (Liu and Peng, 1997). It is evident from this brief overview that learning Chinese and English involves developing a command of distinct word-based and script-relative component skills. In addition, two other characteristics make written Chinese different from the alphabetic writing system of English: (1) diagrams representing different meaningful linguistic units are very common (i.e., morphemes) and (2) graphic elements (e.g., determinatives, classifier, radical) are used to represent the general semantic field of a morpheme (Handel, 2014).

Apart from these characteristics, bilingual children and adults may perform differently in orthography when learning multiple languages. Orthography directly affects word recognition (Diependaele et al., 2013). Monolinguals who must use an orthographic–phonetic working memory strategy instead of a word memory strategy take longer to complete reading tasks, including identifying letter strings that do not exist in the L1 compared to words that follow the orthographic rules of the L1 (Casaponsa et al., 2014). However, bilinguals activate both word recognition systems when presented with one of the languages, suggesting that even completely reliable orthographic cues are not sufficient to limit activation in the target language (Degani et al., 2018).

### Early vs. Late Exposure Effects on Cognitive Skills

Due to the differences in the oral and written components of two distinct languages, such as English and Chinese, one might expect levels of language and cognitive skills to vary with the degree of a bilingual’s exposure to their L2. Indeed, early exposure to the written form of an L2 may change the cognitive functions involved in language learning (Johnson, 2017). Through informal observation and empirical studies, some research evidence supports the notion that early exposure to an L2 is beneficial not only for proficiency in the L2 but also for cognition (Bialystok and Hakuta, 1999; Brito and Barr, 2012). Children with early exposure to an L2 develop a better command of the spoken and written language than those with late exposure. Bilinguals with early L2 exposure are more accurate in their L2 language performance (Kalia et al., 2014). Moreover, early exposure to the L2 may enhance cognitive skills, such as executive functions, because bilingualism keeps both languages active while processing one of them (Bialystok et al., 2004).

However, other studies have shown that early exposure to an L2 is disadvantageous (Sebastián-Gallés et al., 2005; Haman et al., 2017), suggesting that early exposure to an L2 may delay the acquisition of the L1. For example, a study involving bilingual Spanish–Catalan speakers demonstrated that despite early bilingual exposure, participants were unable to distinguish between mispronounced and correctly pronounced words in Spanish and Catalan (Sebastián-Gallés et al., 2005). Even worse, there is research suggesting that early exposure to an L2 has a negative effect on productive grammar in the L1 (Haman et al., 2017). We still do not know whether and to what extent early exposure might be uniquely associated with positive or negative outcomes concerning linguistic and cognitive skills. Likewise, not much is known about the extent to which being exposed to an L1 and an L2 that involve fundamentally different writing systems, such as those of Chinese and English, from an early age might impact cognitive abilities.

### The Impact of Bilingualism on the Cognitive Control Mechanism

Why might being exposed to an L2 through speaking and reading impact cognition? Previous research suggests that early L2 learning and reading may have a positive effect on cognition (e.g., Bialystok and Ryan, 1985; Bialystok et al., 2005) First, bilinguals exercise the brain, thereby improving their performance on nonverbal cognitive tasks, such as attention and working memory (Bialystok, 2017). This bilingual cognitive advantage lies in neuroplasticity, which refers to the ways in which the brain’s abilities to adapt in response to new experiences change throughout a person’s lifetime (Gunnerud et al., 2020). This early advantage has been attributed to bilinguals’ enhanced cognitive control mechanism, which allows them to focus their attention on specific tasks without becoming distracted or retraining habitual behavior (Backer and Bortfeld, 2021).

Additionally, bilingualism can boost the executive function system. The experience of speaking two languages contributes to higher cognitive processes, which operate as a result of more advanced inhibition and attentional abilities (Bialystok and Martin, 2004; Mezzacappa, 2004). The bilingual lexico-semantic system in language processing is regulated by an inhibitory mechanism, which is a solution for cross-language competition. When bilinguals switch from one language to another, they need to suppress the current language and activate the output of another language (Wang et al., 2005). One possible reason for the development of increased cognitive control in bilingual children is that the same control processes are used to solve difficult problems and manage both active language systems (Bialystok et al., 2005).

However, not all research supports a bilingual advantage in cognition. Study of Emmorey et al. (2008a) contradicts the notion of bilingual cognitive enhancement. First, if bilinguals use one language, then their mental representation of the other language is still active and competes with the language currently being used (Marian and Spivey, 2003; Bialystok, 2017). Bilinguals performed worse than their monolingual peers when they were asked to choose their preferred language to answer vocabulary tests (Bialystok, 2017). Because two languages are active all the time, bilinguals must focus on their target language and may be distracted by the other language (Gunnerud et al., 2020). Emmorey et al. (2008b) also examined those bilinguals whose L1 was Cantonese, Italian, or Vietnamese and whose L2 was English. They found that bilinguals did not consistently outperform monolinguals on tasks of cognitive control. The lack of bilingual advantage may be due to differences in motor and perceptual pathways (e.g., visual and auditory pathways) between the two languages, reducing the need for conflict resolution (Emmorey et al., 2008b). It should also be acknowledged that a meta-analysis study based on a large number of published studies gave little support for a bilingual advantage on overall executive function (Gunnerud et al., 2020).

In fact, the specific cognitive advantages and disadvantages of bilingualism may be associated with more language-specific and task-general assessments (Prior and MacWhinney, 2009), structured representations of knowledge (Bialystok and Barac, 2012), and attainment of greater control over working memory (Blom et al., 2014) and attentional procedures (Barac et al., 2014). Thus, considering the extent to which biliteracy skills in Chinese and English facilitate cognitive performance is central to our research topic.

### Rationales for the Hypothesized Research Aims

We therefore provide rationale for our hypothesized research questions. First, regarding the early vs. late exposure to English L2 written literacy, in the present study, *early exposure to English as an L2* refers to children who received formal English reading instruction before age 3. *Late exposure to English as an L2* refers to the students who were first introduced to formal reading instruction in English in grade 3 (*age range*: 8–10 years old). By formal reading instruction, we mean the age at which children were first exposed to the written English language and began to learn to read and write in English in a school setting.

To reiterate, there were two major differences in the degree of English exposure between the early and late groups. First, the onset of formal English instruction for the early group was before age three, but the late group only began to learn English when they entered grade 3. Second, the children in the early-exposure group continued to attend a 90-min individualized after-school English program every day. Both groups attended two compulsory 45-min sessions of English instruction each week, in which the teaching was based on the national-level English curriculum standards. In sum, the two groups differed in the age of onset as well as the overall extent of exposure to English as an L2. Therefore, we first hypothesized that there would be group differences between early vs. late exposure to L2 written literacy, and the extent to which they differed would be dependent upon the relative contributions of the linguistic variables to cognitive variables.

Second, regarding the relative contribution of language, reading and cognitive–linguistic skills to cognitive performance in biliteracy, we categorize the 10 variables into three groups: language only, reading only, and cognitive–linguistic skills. We included receptive and expressive vocabulary in Chinese and English into the language-related skills, word recognition and reading fluency in Chinese and English into the reading-related skills, and phonological awareness and morphological awareness in Chinese (MA-CH) and English into the cognitive–linguistic skills. These factor categories allowed us to investigate the effects of language, reading, and cognitive–linguistic skills on cognitive performance (represented by working memory and attentional control).

We hypothesized that the interrelationship between linguistic and cognitive skills would present differently considering the effect of grade and exposure. More specifically, we predicted that if we put the three categories of language, reading, and cognitive–linguistic skills into a hierarchical regression model in different steps after controlling for intelligence and socioeconomic status (SES), the contribution of these categories should be partitioned out in early elementary school (e.g., grade 3). As age and exposure increase, we predicted that the contribution of language-related biliteracy skills would be positively associated with the cognitive functions of either working memory or attention, or both.

### The Present Study

The goal of the present study was twofold. *First*, as exposure to Chinese–English biliteracy is becoming common and managing two languages might present challenges to cognitive functions, we aimed to examine whether and to what extent early vs. later exposure to Chinese–English biliteracy influences the cognitive processes that are implicated in the process of reading in L1 and L2. *Second*, most previous research has considered working memory as a general ability index that predicts reading and language performance (Baddeley, 1992). A bidirectional approach that examines the common and specific mechanisms underlying the language, reading and cognitive–linguistic interrelationships in working memory is scarce. Our study aimed to address this research gap. Two research questions were concluded below.

**Q1:** Are there differences in linguistic and cognitive skills between Chinese children who in addition to typical exposure to Chinese language and literacy skills, were exposed to English as a second language (L2) and literacy from an early age vs. those who were exposed to English language and literacy later in grade 3?

**Q2:** How do the linguistic-related variables affect cognitive abilities? Specifically, we sought to examine the effects of language skills (vocabulary), reading abilities (word recognition and reading fluency), and cognitive–linguistic skills (phonological awareness and morphological awareness) on cognitive processes (working memory and attention) in Chinese–English bilinguals.

## Materials and Methods

### Participants

A total of 354 students participated in this cross-sectional study, including 178 grade 3 students (88 males, mean age = 9.24 years) and 176 grade 6 students (102 males, mean age = 11.35 years) from different classes at the same school. The school was designated as the experimental site for a series of national-level projects (#31500915, 31571155, 62077011) funded by National Science Funds awarded to the first author from 2015 until 2025. The sample was representative of the population of school-aged children from middle-class families in China according to their family background and SES. The mean family income ranged from 15,000 to 25,000 US dollars, and the average parental educational level was 17.6 years of schooling, i.e., between college and postgraduate education (NIES, 2012). Based on previous research involving this population (e.g., Guan et al., 2014, 2019), participants were typically developing readers and writers. Parents of the children first signed an informed consent form and then completed a background questionnaire of developmental milestones. Two students (0.005% out of the total sample) with a history of neurological or developmental disorders were removed from the study.

The children in the early exposure group had been enrolled in total English immersion programs in nurseries or early learning centers, where formal English learning took place on average for 8 h per day in nurseries and 96 or more class hours per year in early learning centers (Guan et al., 2018). From the time they entered grade 1, these children continued to attend after-school English programs for at least 60 min per day (i.e., 300 min per week). In these after-school programs, they received English exposure in small-group sessions through situated learning, guided observation, or assisted play provided by a native English speaker. The content of instruction included music, fitness, and visual art. The most basic requirements for teachers in both preschool and after-school English programs are a college degree and an English teaching certificate at preschool and primary school.

In contrast, the students in the late exposure group started to learn English in grade 3, which is the official onset time for formal English education in China (Chinese Ministry of Education, 2017). The only difference between the early and late exposure groups was that the children in the late group did not attend the after-school English program. In the current study, this late-exposure group had just started their formal English training when they entered grade 3. The grade 3 children began to learn English at the beginning of the 2019 academic year, and the grade 6 children began their formal English instruction at the beginning of the 2017 academic year. Their exposure to English in school was about 90 min (i.e., two 45-min sessions) per week. They had English exposure in large-sized classes through various teaching methods including task-based, total physical response (Asher, 1966), and communicative approaches (Chinese Ministry of Education, 2017). By the end of grade 6, students’ receptive English vocabulary is expected to be around 3,000 words; by the end of the ninth grade of compulsory education in China, students are expected to have mastered 1,500–1,600 words in English and 200–300 idioms or fixed combinations (Ministry of Education, 2017). Teachers of English in schools are required to possess at least an undergraduate university degree and an English teaching certificate. The children in the early and late groups attended the same schools and were exposed to the same formal English curriculum (i.e., two 45-min sessions of English instruction per week) at school.

### Measures

In a nutshell, we examine the effects of language skills, reading abilities, and cognitive–linguistic skills on cognitive processes in Chinese–English bilinguals. Language skills were measured through receptive vocabulary tests in English and Chinese. Reading skills were investigated through reading fluency tests in English and Chinese and word recognition tasks in both languages. Cognitive–linguistic skills were tested by phonological awareness in English (PA-EN) and Chinese and morphological awareness in English and Chinese. For the dependent variables of cognitive processes, we measured working memory with the Backward Digit Span (BDS), and we tested attention with an attentional control task.

### Cognitive–Linguistic Skills

#### Phonological Awareness in English

A same/different judgment task (Treiman and Zukowski, 1991) was used to assess English PA. This task evaluates phonological awareness at the syllabic, onset-rime, and phoneme levels. Participants judged whether two words share a sound. The experimenter read aloud a pair of words that shared a sound for the following six sets: beginning syllable (*hammer, hammock*), onset (*broom, brand*), initial phonemes (*steak, sponge*), final syllable (*compete, repeat*), rime (*spit, wit*), and final phoneme (*smoke, tack*). There were 10 pairs of items per set, for a total of 60 items. Half of the pairs (*n* = 30) shared a sound, and the other half did not. When the children made five errors in a row, testing stopped. The items were presented by the experimenter at the rate of one item every 5 s.

#### Phonological Awareness in Chinese (PA-CH)

We adapted three judgment tasks to measure Chinese PA (Chen et al., 2010). This task contained both a Mandarin tone awareness task, and a Mandarin onset and rime awareness task. The examiner read three items per set, and the child was asked to judge which one out of the three items did not share the same onset, rime, or tone. For example, the child listened to the words 1/lai4/(赖), 2/lao3/(老), and 3/hai4/(害) and had to indicate that 3/hai4/(害) had a different “beginning” (onset). In this example, the child would circle “3” on the answer sheet. The tone awareness task examined the four tones of Mandarin. The Chinese onset and rime awareness tasks each had 16 sets, and the Mandarin tone awareness task had 16 sets. The items were presented at a rate of 1 every 2 s. The Cronbach’s alpha reliability of this test ranged from 0.83 to.89 across grades 3–6.

#### Morphological Awareness in English (MA-EN)

The morphological awareness task was originally developed by Singson et al. (2000). It contained 20 sentence completion tasks. Children were asked to read each sentence and select on an answer sheet one of four alternative words (or pseudowords) as the correct item that was missing in a sentence. For example, a correct answer for the real word item “He (go, goes, going, gone) to school every day,” was “goes.” For the alternative items “He was (run, ran, running, runned) to school,” the correct answer was “running.” The Guttman split-half reliability coefficient was 0.60 (for real words) and 0.68 (for pseudowords). This was a three-minute task administered in groups.

#### Morphological Awareness in Chinese

A 10-min oral performance task was used to assess morphological awareness in Chinese. This morphological compound task (Guan et al., 2015) consisted of two parts: one focusing on generating left-headed two-character compounds [i.e., the first character of all compounds is the same, like the left-headed character 马 (*horse*) in the two-character compound 马车 (*horse-driven cart*) or 马头 (*horse head*)], and one focusing on generating right-headed two-character morphological compound words with eight base items each [i.e., the second character of all compounds are the same, like the right-headed character 马 (*horse*) in the two-character compound 上马 (*get on the horse*) or 骑马 (*ride the horse*)]. Students could choose any six base forms to produce as many “right-headed” two-character words as they could within 5 min. They were then asked to choose any six base forms to produce as many “left-headed” two-character words in another 5 min. One of the base forms was “马” (ma3 “horse”). The participants were required to generate as many compound words as possible that included the given base form, including 马上 (*immediately*), 马路 (*road*). The students’ oral performance was audio-taped and was scored by two research assistants. Inter-rater reliability was 0.97. Cronbach alpha internal consistency reliability of this task ranged from 0.75.to 0.95 in grades 3 to 6.

### Reading Abilities

#### Reading Fluency in English (RF-EN) and Chinese (RF-CH)

The Test of Word Reading Efficiency (TOWRE; Torgesen et al., 2012) was used to assess reading fluency. We adapted the English version of the TOWRE task to create a Chinese version (Guan et al., 2019). The scores of this sentence reading fluency (RF) task in both Chinese and English were based on the number of syllables read correctly per minute. This was done to enable comparisons between Chinese and English on the same RF metric. There were 100 items in total in both Chinese and English. Testing was stopped after 3 min. In each language, the score was the number of items read correctly in 3 min.

#### Word Recognition in English (Word Recog-EN) and in Chinese (Word Recog-CH)

The English and Chinese word recognition measures involved lexical decision tasks (Guan and Fraundorf, 2020a; Guan et al., 2020b). To select materials for the lexical decision task in Chinese, we randomly sampled 240 characters (40 from each grade level from grade 1 to grade 6) from the standard curriculum, ensuring that the items were representative of the compound regularities and configurations of Chinese characters. The basic configurations include left–right, top–down, and outside–inside. We defined characters as high consistency if the semantic radical appeared with the same pronunciation in more than 50% of the characters (Shu and Anderson, 1999) and low consistency if not, and we used the curricular grade level as a proxy for age of acquisition. Another 240 pseudo-characters were created by adding, deleting, or shifting one stroke from the radicals within a legal character.

To select materials for the lexical task in English, we randomly sampled 240 words (40 from each grade level from grade 1 to grade 6) from the national English curriculum, ensuring that the testing items were representative of the letter–sound consistency, frequency of English words, and word reading level from each of the six grades. Again, we took the curricular grade level as a proxy for age of acquisition. Another 240 pseudowords were created by changing the onset, syllable, or rime of the real words; by swapping the letter order within a word; or by changing a single letter or a cluster of letters within a word.

The children received a practice trial to familiarize them with these two word recognition tasks and then moved on to the real testing session, in which they judged whether each of the 480 words was real or not, one at a time; reaction time (RT) and accuracy were recorded by computer. The order of administering the word recognition tasks in Chinese and English was counterbalanced.

### Language Skills

#### Receptive Vocabulary in English (Rec-Voc-EN) and in Chinese (Rec-Voc-CH)

Vocabulary was assessed using a standardized test of receptive vocabulary (Peabody Picture Vocabulary Test; PPVT-IV; Dunn and Dunn, 2007). It was an individualized assessment in both English and the adapted version in Chinese (Guan et al., 2014). For each item, a card consisting of four pictures was shown. The students were required to identify the picture that matched the word said by the experimenter. The difficulty level of the vocabulary presented increased gradually. The test contained 90 multiple-choice vocabulary items in English and 90 in Chinese. The presentation order in either English or Chinese was counterbalanced. The students were required to match words that they heard with the corresponding pictures. It took 10 min to complete the test. One point was awarded for each correct answer and the maximum score was 90. The children were required to circle the picture that matched the word they heard.

### Cognitive Skills

#### Working Memory: Backward Digit Span

The Backward Digit Span was used to assess working memory (Hester et al., 2004; Laures-Gore et al., 2011). The central executive component of working memory has been argued to play a crucial part in the performance of span tasks, especially backward span (Hester et al., 2004). The task consisted of 10 sets of randomly presented single digits. The number of digits per set gradually increased from 5 to 9, and the students were expected to write the digits they heard in reverse order. For example, when listening to the digits 4, 9, 1, 3, 7 said by the experimenter, the participants were required to write these digits in a backward order (7, 3, 2, 9, 4). There were 10 items in this task and all items were administered. The total testing time for this test was 2 min. Scores ranged from 0 to 10, and the maximum score was 10. This task was a group-administered test. Instructions were given in Chinese, but the students were required to write Arabic numerals (i.e., 1–10).

#### Attentional Control

The Simon task was used to assess attentional control. It was group-administered. Each individual student performed the task on their own computer screen. The researchers guided all the students to do this task in the same computer room. The stimuli were presented on a 15-inch color monitor of a Dell laptop with a white background. Participants sat approximately 60 cm away from the screen and placed their index fingers of each hand gently on the left and right shift keys, respectively. These keys were labeled with white stickers displaying a circle for the left shift key and a cross for the right shift key to make it easy to explain the rules. The attentional control (Simon) task was programmed by E-prime 2.0. Each trial began with fixation in the center of the screen that remained visible for 800 ms, followed by a 250-ms blank interval. After this, a stimulus appeared on the left or right side of the screen and remained on the screen for 1,000-ms if there was no response. The positions for X and Y were computed from the origin (X = 0, Y = 0) on the bottom left of the display. The clock began with the onset of the stimulus, and the response terminated the stimulus. There was a 500-ms blank interval before the onset of each trial. The children were required to press the left shift key when the arrow pointed left and the right shift key when the arrow pointed right.

The administration of the attentional control (Simon) task began with a practice block of 20 trials and proceeded with 120 experimental trials. Both the accuracy and response times were recorded. Since the mean accuracy was above 0.92, only the response times for congruent and incongruent trials were used for the final analysis. The attentional control task was scored by subtracting the response time in the congruent condition from that in the incongruent condition.

Descriptive statistics and Cronbach’s alpha reliabilities for each grade and early/late group comparisons for all measures are summarized in Table 1.

**Table 1:** Intercorrelations among English and Chinese cognitive, linguistic, and reading tasks: grade 3 (*N* = 178) below the diagonal and grade 6 (*N* = 176) above the diagonal.

	1	2	3	4	5	6	7	8	9	10	11	12
1. WM-BDS	–	0.174^*^	−0.042	0.091	0.163^*^	0.202^**^	0.009	0.136	0.122	−0.042	0.153^*^	0.198^**^
2. Att Control	0.172^*^	–	0.017	−0.080	−0.105	−0.148	−0.104	−0.078	−0.029	−0.143	−0.139	−0.221^**^
3. PA-EN	0.299^**^	0.100	–	0.125	0.231^*^	−0.134	0.251^*^	0.111	0.202^**^	0.164^*^	0.050	0.023
4. PA-CH	0.260^**^	0.181^*^	0.305^**^	–	0.167^*^	0.182^*^	0.084	0.375^**^	0.076	0.224^**^	0.381^**^	0.449^**^
5. MA-EN	0.019	−0.077	−0.103	0.016	–	0.153^*^	0.279^*^	−0.001	0.187^**^	−0.073	0.281^**^	0.241^**^
6. MA-CH	−0.061	0.073	0.162^*^	0.290^*^	−0.155^*^	–	−0.007	0.128^*^	0.108	0.241^**^	−0.005	0.216^*^
7. RF-EN	0.171^*^	0.038	0.310^*^	0.373^**^	−0.032	0.043	–	0.249^**^	0.314^*^	−0.018	0.197^**^	0.208^**^
8. RF-CH	0.351^**^	0.133	0.269^**^	0.428^**^	−0.072	−0.005	0.433^**^	–-	0.113	0.236^**^	0.372^**^	0.471^**^
9. Word Recog-EN	−0.031	0.141	0.029	0.037	−0.113	0.264^**^	0.043	0.068	–	0.234^**^	0.282^**^	0.049
10. Word Recog-CH	−0.073	−0.159	−0.121	0.060	−0.057	0.216^*^	0.085	−0.107	0.103	–	0.033	0.311^**^
11. Rec-Voc-EN	0.338^**^	0.293^**^	0.234^**^	0.238	−0.077	0.142	0.425^**^	0.278	0.193^**^	0.185^*^	–	0.428^**^
12. Rec-Voc-CH	0.164^*^	0.266^**^	−0.036	0.370^**^	−0.008	0.025	0.106	0.368^*^	0.370	0.076	0.182^*^	

WM BDS, Backward Digit Span; Att Control, Attentional Control Simon Task; PA-EN, Phonological Awareness in English; PA-CH, Phonological Awareness in Chinese; MA-EN, Morphological Awareness in English; MA-CH, Morphological Awareness in Chinese; RF-EN, text Reading Fluency in English; RF-CH, text Reading Fluency in Chinese; Word Recog-EN, Word Recognition in English; Word Recog-CH, Word Recognition in Chinese; Rec-Voc-EN, Receptive Vocabulary in English; Rec-Voc-CH, Receptive Vocabulary in Chinese. The values for grade 3 are shown below the diagonal. The values for grade 6 are shown above the diagonal.

* Correlation is significant at the 0.05 level (2-tailed).

** Correlation is significant at the 0.01 level (2-tailed).

### Procedures

The Beijing Language and Culture University (BLCU) ethics committee approved the study. Parents of the children in grades 3 and 6 signed the informed consent form and completed a background screening survey to ensure that the students recruited for the study had no neurological disorders, mental disabilities, or learning difficulties. The parents provided their annual family income and completed an online family and language learning background survey, which asked about their children’s basic language learning and usage experience.

Data collection took place in mid-October (i.e., the beginning of the academic year of 2019 for grades 3 and 6). We chose grade 3 because the late-exposure group had just begun to receive formal English instruction in grade 3. The word reading task was administered individually. All the other tasks were group-administered to whole classrooms. There were 2 classrooms of children with early exposure to English currently receiving after-school English instruction and another 2 classrooms of children who were first exposed to English in grade 3 receiving only regular in-school English instruction. Students’ general intelligence scores were also assessed by Raven’s Matrices at the beginning of the academic school year. The average class size ranged between 40 and 50 students. Instructions for each task were audio-taped and played to the participants so that all the tasks were administered uniformly across groups. Instructions for the English tasks were provided in Chinese.

### Data Analyses

We conducted hypothesis tests to address the two sets of major research questions. The data were analyzed in the following steps. First, two separate Pearson correlation analyses for each grade were conducted with three different measures of reading, language, and cognitive–linguistic abilities. Descriptive statistics were used to examine the basic data distribution of all variables. Second, a multivariate analysis of variance (MANOVA) was conducted to test the main effect of exposure (early vs. late) as well as the interaction effects between early vs. late exposure and all related linguistic measures on cognitive skill performance. Finally, hierarchical regression analyses of three models for each grade were used to uncover the relative contributions of linguistic measures (reading, language, and cognitive–linguistic skills) to the cognitive abilities of working memory and attentional control.

The descriptive statistics focused on two independent variables: early vs. late exposure to biliteracy and grade effects (grade 3 and grade 6). There was no ceiling or floor effect for any of the measure in either Chinese or English. Outliers were defined as observations above or below 2.5 SD of the mean of that measure for each grade. Overall, this resulted in 5 percent of individual grade-level data being excluded as outliers, within the 5 to 10% recommended by Ratcliff (1993).

There were moderate-to-high correlations between the same measures over time. The correlations were based on full information maximum likelihood estimation (e.g., McArdle, 1994), an estimation method that allowed for the examination of sample descriptive statistics as if all members of the sample were present at all measurement occasions. We compared these correlations based on full information maximum likelihood as well as the means and standard deviations with the typical correlations based on pairwise deletions and with all available data (see Pigott, 2001, for a review).

## Results

### Correlation and Descriptive Analyses

Results of analyses concerning the first research question, namely whether there are differences in language, reading, and cognitive–linguistic skills, between the early-exposure and late-exposure groups are summarized in Tables 1–3. Table 1 presents the correlation coefficients among all the measures in the study. The main results obtained from the correlation analyses indicate that overall, the linguistic measures (e.g., PA, Vocab) were all positively correlated with one another in both grades 3 and 6, showing the uniform development of linguistic performance across the elementary years. Some cognitive measures (e.g., attention) were correlated with reading-related measures (e.g., RF) in grade 3 but not in grade 6, suggesting that there might be some differences in the relationship between cognition and reading development between the younger (grade 3) and older students (grade 6); the cognitive–linguistic measures (e.g., MA) were not correlated with reading measures (e.g., word recognition) in grade 3, but were positively correlated in grade 6, suggesting that, as age increases, the relative contribution of linguistic and cognitive measures to reading might change.

**Table 2:** Descriptive statistics by tasks for grade 3: early vs. late exposure to English.

	Early exposure (*N* = 86)		Late exposure (*N* = 64)		*p*-value	*ƞ*
	*M* (SD)	Cronbach’s *α*	*M* (SD)	Cronbach’s *α*		
Raven’s Matrices	23.12 (4.1)	0.86	24.5 (4.8)	0.83	0.52	0.12
*Language*						
Rec-Voc-EN	35 (16)	0.78	32 (18)	0.84	0.11	0.004
Rec-Voc-CH	37 (9)	0.80	42 (13)	0.81	0.05^*^	0.013
*Reading*						
Word Recog-EN	0.59 (0.17)	0.82	0.59(0.18)	0.82	0.46	0.001
Word Recog-CH	0.58 (0.11)	0.81	0.59(0.12)	0.83	0.76	0.001
RF-EN	18 (12)	0.82	14 (11)	0.81	0.43	0.001
RF-CH	24 (10)	0.80	25 (10)	0.85	0.66	0.001
*Cognitive–Linguistic*						
PA-EN	14.3 (2)	0.79	14.1 (3)	0.82	0.66	0.001
PA-CH	24.2 (8)	0.81	25.4 (8)	0.82	0.02^*^	0.005
MA-EN	8.2 (1)	0.83	8.1 (1)	0.82	0.50	0.003
MA-CH	3.1 (1)	0.82	3.8 (1)	0.83	0.01^*^	0.013
*Cognitive abilities*						
WM-BDS	4 (2)	0.79	3 (1.7)	0.83	0.032^*^	0.033
Att Control	−6 (38)	0.80	−6 (41)	0.85	0.987	0.000

Standard deviations are given in parentheses. Language was measured using Receptive Vocabulary. Reading was measured using text reading fluency and word recognition, cognitive–linguistics skills were measured using Phonological Awareness and Morphological Awareness. These above-mentioned measures were assessed in both Chinese and English. Cognitive abilities were measured using backward Digit Span and the Attentional Control Simon task. WM BDS, Backward Digit Span; Att Control, Attentional Control Simon Task; PA-EN, Phonological Awareness in English; PA-CH, Phonological Awareness in Chinese; MA-EN, Morphological Awareness in English; MA-CH, Morphological Awareness in Chinese; RF-EN, text Reading Fluency in English; RF-CH, text Reading Fluency in Chinese; Word Recog-EN, Word Recognition in English; Word Recog-CH, Word Recognition in Chinese; Rec-Voc-EN, Receptive Vocabulary in English; Rec-Voc-CH, Receptive Vocabulary in Chinese, *stands for significant at 0.05 level.

**Table 3:** Descriptive statistics by tasks for grade 6: early vs. late exposure to English.

	Early exposure (*n* = 96)		Late exposure (*n* = 76)		*p*-value	*ƞ*
	*M* (SD)	Cronbach’s *α*	*M* (SD)	Cronbach’s *α*		
Raven’s Matrices	36.1 (5.3)	0.86	35.5 (5.1)	f0.83	0.67	0.08
*Language*						
Rec-Voc-EN	78.0 (11)	0.78	72 (12)	0.92	0.022^*^	0.030
Rec-Voc-CH	75.0 (15)	0.82	74 (17)	0.83	0.181	0.008
*Reading*						
Word Recog-EN	0.72 (0.15)	0.85	0.66(0.18)	0.87	0.044^*^	0.034
Word Recog-CH	0.81 (0.14)	0.83	0.79(0.13)	0.88	0.316	0.006
RF-EN	33.6 (5)	0.87	27.5 (5)	0.89	0.035^*^	0.025
RF-CH	32.2 (5.8)	0.86	30.5 (8.1)	0.88	0.079	0.005
*Cognitive–Linguistic*						
PA-EN	16 (1.9)	0.90	17 (2.1)	0.91	0.753	0.001
PA-CH	47 (9.8)	0.88	46 (9.9)	0.85	0.098	0.025
MA-EN	8.9 (1.2)	0.86	8.5 (1.5)	0.95	0.031^*^	0.027
MA-CH	5.7 (2.6)	0.90	5.4 (2.7)	0.90	0.351	0.007
*Cognitive abilities*						
WM-BDS	7.8 (2.4)	0.92	6.6 (2.9)	0.92	0.008^*^	0.039
Att Control	8.0 (2.2)	0.87	3.0 (1.1)	0.88	0.283	0.007

Standard deviations are given in parentheses. Language was measured using receptive Vocabulary. Reading was measured using text reading fluency and word recognition, cognitive–linguistics skills were measured using Phonological Awareness and Morphological Awareness. These above-mentioned measures were assessed in both Chinese and English. Cognitive abilities were measured using backward Digit Span and the Attentional Control Simon task. WM BDS, Backward Digit Span; Att Control, Attentional Control Simon Task; PA-EN, Phonological Awareness in English; PA-CH, Phonological Awareness in Chinese; MA-EN, Morphological Awareness in English; MA-CH, Morphological Awareness in Chinese; RF-EN, text Reading Fluency in English; RF-CH, text Reading Fluency in Chinese; Word Recog-EN, Word Recognition in English; Word Recog-CH, Word Recognition in Chinese; Rec-Voc-EN, Receptive Vocabulary in English; Rec-Voc-CH, Receptive Vocabulary in Chinese, *stands for significant at 0.05 level.

Tables 2 and 3 present the descriptive statistics and Cronbach’s alpha reliabilities of all measures for both early exposure and late exposure in grades 3 and 6. In grade 3, the early exposure to English group performed worse than the late exposure group on two Chinese cognitive–linguistic tasks, morphological awareness (*p* < 0.01), and phonological awareness (*p* = 0.02), but outperformed the late-exposure group on working memory (*p* = 0.032). There were no significant differences between the early and late groups on the other tasks in grade 3.

In grade 6, the early-exposure group outperformed the late-exposure group on four measures: English receptive vocabulary (*p* = 0.022), word recognition (*p* = 0.044), reading fluency (*p* = 0.035), and working memory (*p* = 0.008). Overall, in both grade 3 and grade 6, the early-exposure groups performed better on working memory than the late-exposure groups.

### Multivariate Analyses of Variance

A MANOVA was performed to address the second research question concerning the role that linguistic variables play in cognitive abilities between the early- and late-exposure groups. The MANOVA results revealed different patterns of exposure effects, grade effects, and linguistic variable effects, and all combinations of exposure by linguistic interaction effects on working memory and attentional control. We built all combinations of interactive terms with early vs. late exposure. This resulted in over 40 product terms and their corresponding effects, but only two product terms involving word recognition in Chinese and morphological awareness in Chinese were revealed to be significant. To save space, we report only the significant multivariate interaction effects in Table 4.

**Table 4:** Significant multivariate main effects and interaction of exposure (early/late) to English and grade on working memory and attentional control (at *p* < 0.001 level).

Source	Dependent variable	*df*	*F*	*p*-value	*ƞ*
Exposure	Working memory	1/291	8.425	0.004	0.028
	Attentional control	1/291	0.711	0.400	0.002
Grade (3; 6)	Working memory	1/291	155.867	<0.001	0.336
	Attentional control	1/291	9.077	0.003	0.029
Exposure × MA-CH	Working memory	2/291	11.016	<0.001	0.071
	Attentional control	2/291	0.162	0.651	0.001
Exposure × Word Recog-CH	Working memory	2/291	1.392	0.250	0.010
	Attentional control	2/291	7.157	0.006	0.075

Exposure, early vs. late exposure; MA-CH, morphological awareness in Chinese; Word Recog-CH, word recognition in Chinese.

As can be seen from Table 4, there was a significant effect of age of exposure to written English on working memory (*p* = 0.004), a significant effect of grade (*p* < 0.001) on working memory, and a significant effect of grade on attentional control (*p* = 0.003). There was a significant interaction effect of age of exposure by morphological awareness in Chinese (*p* = 0.007) on working memory. There was also a significant interaction effect of age of exposure × word recognition in Chinese (*p* = 0.007) on attention. None of the interaction terms involving grade effect (3 vs. 6) were significant.

### Hierarchical Regression Analyses

As the main purpose of the present study is to investigate linguistic influences on cognition, we only present the general pattern of relative contribution of linguistic variables to the cognitive skill performance. Therefore, three orders of hierarchical regression analyses were conducted for each grade, respectively. We put three categories of linguistic variables in three different orders, respectively, adding exposure (early vs. late) in the last step. After controlling for general nonverbal ability and SES, the order of variables entered in Model 1 was language, reading, cognitive–linguistic skills, and the categorical variable “exposure” (early/late). The order for variables in Model 2 was cognitive–linguistic skills, followed by language, reading, and exposure. The order for Model 3 was reading, cognitive–linguistic skills, language, and exposure. Tables 5 and 6 display the results of these hierarchical regression analyses in which we regressed cognitive skills on unique reading, language, and cognitive–linguistic variables in grades 3 and 6, respectively. Results on the left pertain to working memory as the dependent variable, and results on the right pertain to attention as the dependent variable.

**Table 5:** *R*^2^ change in each block of predictors in three models using hierarchical multiple regression switch-order approach to explain variance on two measures of cognitive skills in grade 3.

Models	Step	Models	Working memory			Attentional control		
			*R* ^2^	*R*^2^ change	*p*-value	*R* ^2^	*R*^2^ change	*p*-value
Model 1	1.	Nonverbal ability	0.131	0.131	<0.001	0.080	0.080	<0.001
	2.	Language	0.256	0.125	<0.001^*^	0.127	0.127	<0.001^*^
	3.	Word Reading	0.324	0.066	0.009^*^	0.190	0.062	0.042^*^
	4.	Cognitive–Linguistic	0.366	0.043	0.057	0.194	0.004	0.995
Model 2	1.	Nonverbal ability	0.131	0.131	<0.001	0.080	0.080	<0.001
	2.	Cognitive–Linguistic	0.266	0.135	<0.001^*^	0.042	0.042	0.217
	3.	Language	0.360	0.094	0.001^*^	0.143	0.101	0.005^*^
	4.	Word Reading	0.366	0.006	0.517	0.194	0.051	0.019^*^
Model 3	1.	Nonverbal ability	0.131	0.131	<0.001	0.080	0.080	<0.001
	2.	Word Reading	0.277	0.146	<0.001^*^	0.101	0.101	0.006^*^
	3.	Cognitive–Linguistic	0.340	0.064	0.010^*^	0.115	0.014	0.724
	4.	Language	0.390	0.050	0.004^*^	0.200	0.085	0.001^*^

*stands for significant at 0.05 level.

**Table 6:** *R*^2^ change of each block of predictors in three models using hierarchical multiple regression switch-order approach to explain variance on two measures of cognitive skills in grade 6.

Models	Step	Models	Working memory			Attentional control		
			*R* ^2^	*R*^2^ change	*p*-value	*R* ^2^	*R*^2^ change	*p*-value
Model 1	1.	Variable controlled	0.09	0.090	<0.001	0.080	0.080	<0.001
	2.	Language	0.048	0.039	0.031^*^	0.050	0.050	0.013^*^
	3.	Reading	0.0153	0.024	0.376	0.082	0.032	0.229
	4.	Cognitive–Linguistic	0.197	0.044	0.093	0.097	0.015	0.612
Model 2	1.	Variable controlled	0.09	0.090	<0.001	0.080	0.080	<0.001
	2.	Cognitive–Linguistic	0.152	0.062	0.026^*^	0.032	0.032	0.245
	3.	Language	0.17	0.018	0.189	0.067	0.035	0.047^*^
	4.	Reading	0.322	0.027	0.301	0.097	0.030	0.263
Model 3	1.	Variable controlled	0.09	0.090	<0.001	0.080	0.080	<0.001
	2.	Reading	0.252	0.162	<0.001^*^	0.029	0.029	0.001^*^
	3.	Cognitive–Linguistic	0.319	0.067	<0.001^*^	0.040	0.011	0.123
	4.	Language	0.324	0.005	0.101	0.045	0.005	0.186

* *p* < 0.05;

** stands for significant at 0.10 level.

As can be seen in Table 5, in grade 3, reading measures (i.e., word recognition and reading fluency in both Chinese and English) jointly explained almost 13% of the variance in working memory (*p* < 0.001); vocabulary explained an additional 6.4% of the variance in working memory (*p* < 0.001); and cognitive–linguistic variables (i.e., phonological awareness and morphological awareness) explained an additional 5% of the variance in working memory (*p* = 0.001). As for the second dependent variable—attentional control—reading variables explained over 10% of the variance, cognitive–linguistic variables explained almost 12% of the variance, and language (i.e., receptive vocabulary in both Chinese and English) explained almost 13% of the variance on attentional control (*p* = 0.001).

As can be seen in Table 6, as for grade 6, Model 3 seems to be the best model as the reading variables explained over 16% of the variance significantly, cognitive–linguistic variables explained almost 7% of the variance significantly, and language (vocabulary) contributed 1% of the variance in working memory (*p* < 0.001). As for attentional control, only vocabulary was significant (*p* < 0.001), explaining 5% of the variance. These results suggest that, by grade 6, when children’s language skills in English and Chinese reached a certain level of proficiency, the impact of early onset of biliteracy skills on cognitive functions such as attention was not discernable.

As seen in Tables 7 and 8, English vocabulary and phonological awareness in English were significantly associated with working memory in grade 3. Word recognition in Chinese and vocabulary in Chinese were significantly associated with attention in grade 3. Morphological awareness in Chinese was significantly associated with working memory for grade 6. Word recognition in English was significantly associated with attention in grade 6.

**Table 7:** The contribution of SES, intelligence, reading and linguistic measures to cognitive skills at grade 3—hierarchical regression summary table.

Models	Steps	Variables	Working memory			Attentional control		
			Beta	*t*	*p*-Value	Beta	*t*	*p*-Value
Model 1	1. SES & Intelligence	Control variables	0.213	0.967	0.068	0.211	0.969	0.078
	2. Language	Recep-Vocab-EN	0.221	2.456	0.015^*^	0.221	2.456	0.015^*^
		Recep-Vocab-CH	0.137	1.960	0.052	0.137	1.960	0.004
	3. Reading	Word Recog-EN	−0.064	−0.883	0.378	−0.064	−0.883	0.833
		Word Recog-CH	−0.051	−0.707	0.480	−0.051	−0.707	0.005
		RF-EN	−0.032	−0.388	0.698	−0.032	−0.388	0.266
		RF-CH	0.191	2.248	0.026^*^	0.191	2.248	0.862
	4. Cognitive–linguistic	PA-EN	0.214	2.850	0.005^*^	0.214	2.850	0.966
		PA-CH	0.001	0.006	0.995	0.001	0.006	0.848
		MA-EN	0.008	0.113	0.910	0.008	0.113	0.611
		MA-CH	0.104	−0.143	0.155	0.104	−0.143	0.654
Model 2	1. SES & Intelligence	Control variables	0.218	0.962	0.075	0.215	0.957	0.085
	2. Cognitive–linguistic	PA-EN	0.214	2.850	0.005^*^	0.004	0.042	0.966
		PA-CH	0.001	0.006	0.995	0.020	0.192	0.848
		MA-EN	0.008	0.113	0.910	−0.043	−0.510	0.611
		MA-CH	−0.104	−1.430	0.155	0.040	0.450	0.654
	3. Language	Recep-Vocab-EN	0.191	2.248	0.026^*^	0.017	0.174	0.862
		Recep-Vocab-CH	−0.032	−0.388	0.698	−0.106	−1.117	0.266
		Word Recog-EN	0.221	2.456	0.015^*^	0.272	1.476	0.055
	4. Reading	Word Recog-CH	0.137	1.960	0.052	0.246	2.895	0.004^*^
		RF-EN	−0.064	−0.883	0.378	0.0191	0.2111	0.833
		RF-CH	−0.051	−0.707	0.480	−0.237	−2.857	0.005^*^
Model 3	1. SES & Intelligence	Control variables	0.217	0.967	0.082	0.223	0.978	0.082
	2. Reading	Word Recog-EN	−0.120	−1.600	0.111	−0.009	−0.095	0.924
		Word Recog-CH	−0.082	−1.147	0.253	−0.244	−2.957	0.004^*^
		RF-EN	0.192	1.379	0.065	0.146	1.506	0.135
		RF-CH	0.189	1.340	0.068	−0.004	−0.044	0.965
		PA-EN	0.256	3.398	0.001^**^	0.048	0.528	0.599
	3. Cognitive–linguistic	PA-CH	−0.076	−0.856	0.393	−0.058	−0.559	0.577
		MA-EN	−0.031	−0.440	0.661	−0.058	−0.689	0.492
		MA-CH	−0.117	−1.634	0.104	0.027	0.305	0.761
	4. Language	Recep-Vocab-EN	0.211	2.402	0.017^*^	0.231	1.140	0.054
		Recep-Vocab-CH	0.144	2.093	0.038	0.233	2.737	0.004^*^

PA-EN, Phonological Awareness in English; PA-CH, Phonological Awareness in Chinese; MA-EN, Morphological Awareness in English; MA-CH, Morphological Awareness in Chinese; RF-EN, text Reading Fluency in English; RF-CH, text Reading Fluency in Chinese; Word Recog-EN, Word Recognition in English; Word Recog-CH, Word Recognition in Chinese; Recep-Vocab-EN, Receptive Vocabulary in English; Recep-Vocab-CH, Receptive Vocabulary in Chinese.

* *p* < 0.05;

** *p* < 0.01.

**Table 8:** The contribution of SES, intelligence, reading, and linguistic measures to cognitive skills in grade 6—hierarchical regression summary table.

Models	Variables	Working memory			Attentional control		
		Beta	*t*	*p*-Value	Beta	*t*	*p*-Value
1. SES & Intelligence	Variable controlled	0.215	0.958	0.084	0.211	0.965	0.082
2. Language	Recep-Vocab-EN	−0.003	−0.024	0.981	−0.002	−0.014	0.989
	Recep-Vocab-CH	0.146	1.208	0.229	−0.237	−1.897	0.060
	Word Recog-EN	0.114	1.391	0.166	0.062	3.741	0.006^**^
3. Reading	Word Recog-CH	−0.098	−1.225	0.222	−0.159	−1.957	0.052
	RF-EN	−0.047	−0.565	0.573	−0.102	−1.201	0.232
4. Cognitive–linguistic	RF-CH	0.106	1.224	0.223	0.011	0.117	0.907
	PA-EN	−0.028	−0.364	0.717	0.019	0.235	0.814
	PA-CH	−0.053	−0.540	0.590	0.088	0.883	0.378
	MA-EN	0.082	1.018	0.310	−0.030	−0.360	0.719
	MA-CH	0.181	2.273	0.024^*^	−0.093	−1.143	0.255
1. SES & Intelligence	Variable controlled	0.214	0.968	0.086	0.218	0.978	0.085
2. Cognitive–linguistic	PA-EN	−0.028	−0.364	0.717	0.019	0.235	0.814
	PA-CH	−0.053	−0.540	0.590	0.088	0.883	0.378
	MA-EN	0.082	1.018	0.310	−0.030	−0.360	0.719
	MA-CH	0.181	2.273	0.024	−0.093	−1.143	0.255
3. Language	Recep-Vocab-EN	−0.003	−0.024	0.981	−0.002	−0.014	0.989
	Recep-Vocab-CH	0.146	1.208	0.229	−0.237	−1.897	0.060
	Word Recog-EN	0.114	1.391	0.166	0.062	0.741	0.460
4. Reading	Word Recog-CH	−0.098	−1.225	0.222	−0.159	−1.957	0.042^*^
	RF-EN	−0.047	−0.565	0.573	−0.102	−1.201	0.232
	RF-CH	0.106	1.224	0.223	0.011	0.117	0.907
1. SES & Intelligence	Variable controlled	0.216	0.957	0.084	0.215	0.964	0.072
2. Reading	Word Recog-EN	0.079	2.236	0.026	0.138	3.325	0.001
	Word Recog-CH	0.000	0.006	0.995	−0.043	−1.008	0.314
	RF-EN	0.062	1.643	0.101	0.021	0.486	0.627
	RF-CH	0.158	3.868	0.001^**^	−0.077	−1.632	0.103
3. Cognitive–linguistic	PA-EN	0.021	0.591	0.555	0.041	0.962	0.336
	PA-CH	0.137	2.572	0.010^*^	0.052	0.831	0.406
	MA-EN	0.081	2.328	0.020^*^	0.020	0.490	0.625
4. Language	MA-CH	0.097	2.629	0.009^**^	0.021	0.479	0.632
	Recep-Vocab-EN	0.089	1.649	0.100	0.116	1.787	0.074
	Recep-Vocab-CH	0.035	0.742	0.458	−0.061	−1.054	0.292

PA-EN, Phonological Awareness in English; PA-CH, Phonological Awareness in Chinese; MA-EN, Morphological Awareness in English; MA-CH, Morphological Awareness in Chinese; RF-EN, text Reading Fluency in English; RF-CH, text Reading Fluency in Chinese; Word Recog-EN, Word Recognition in English; Word Recog-CH, Word Recognition in Chinese; Recep-Vocab-EN, Receptive Vocabulary in English; Recep-Vocab-CH, Receptive Vocabulary in Chinese.

* *p* < 0.05;

** *p* < 0.01.

To test the effects of linguistic variables on cognitive performance for early exposure to English, we examined coefficient estimates in greater depth. The results for grade 3 and grade 6 are summarized in Tables 7 and 8, respectively. Two key results are notable. First, morphological awareness in Chinese was a significant predictor of working memory (*p* < 0.001). Second, word recognition in Chinese had a significant effect on attention (*p* = 0.006). Including early vs. late exposure into the hierarchical modeling did not affect the results. That is, the general patterns of the contributions of linguistic skills to cognitive abilities were stable, regardless of the degree of L2 exposure.

The first question addressed in this study was whether there are differences in linguistic and cognitive skills between the early- and late-exposure groups. Results indicated that early simultaneous exposure to Chinese (L1) and English (L2) language and literacy skills starting at the age of 3 leads to a temporary delay in students’ performance on Chinese morphological awareness, phonological awareness, and vocabulary in third grade, in comparison with students who started to learn English later. The children with concurrent early exposure to Chinese and English pay a price. However, this delay is temporary, and in subsequent years, with continued systematic exposure to language and literacy skills in the L1 and the L2, the early-exposure group catches up to the late-exposure group on their Chinese skills. Moreover, by grade 6, the performance of the early-exposure group in working memory is superior to that of the late-exposure group, though this advantage is not evident on attention control, the second cognitive skill under study.

Second, MANOVA results show that there was an interaction of exposure (early/late) by scriptal-specific effect, namely the interaction between the linguistic features and learner characteristics (Guan et al., 2020b) but not grade by scriptal effect. This suggests that the effects of linguistic skills (e.g., vocabulary) on cognitive skill performance (e.g., working memory and attention) were mediated by exposure (early vs. late) but not by age. Only the effects of morphological awareness and word recognition in Chinese on working memory are associated with the degree of exposure.

Third, there was a general scriptal effect, namely the association between sensitivity to the orthographic form of the native language and general cognitive function. This suggests that morphological awareness in Chinese was significantly associated with working memory, whereas word recognition in Chinese was associated with attention for the younger (grade 3) Chinese–English bilinguals. Notably, regardless of early or late exposure to English, morphological awareness and word recognition in English were significantly related to working memory in grade 6, confirming that performance on these linguistic and reading tasks is generally related to working memory. At the same time, the distinction between early and later exposure to English did not play a role in the results of the regression analyses.

**Figure 1:**
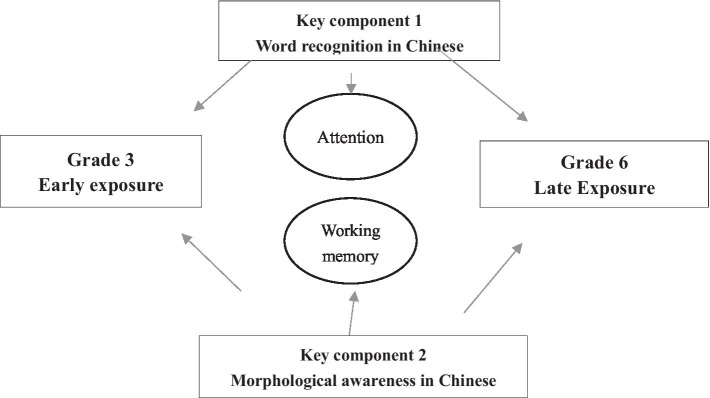
Conceptual framework of effects of linguistic, reading, and cognitive–linguistic abilities on cognitive abilities. Q2 How do the linguistic-related variables affect cognitive abilities?

Results of two research questions are concluded and displayed in Table 9 and Figure 1.

**Table 9:** Summary of the major findings.

Exposure	Grade 3	Grade 6
	Early vs. Late	Early vs. Late
Working memory	**>**	**>**
Morphological awareness	**<**	ns
Phonological awareness	**<**	ns
English receptive vocabulary task (linguistic abilities)	ns	**>**
Word recognition	ns	**>**
Reading fluency (reading abilities)	ns	**>**

Q1 Difference in linguistic and cognitive skills.

We summarize the major findings: (1) According to Table 9, there was a temporary delay in students’ performance on Chinese morphological awareness, phonological awareness, and vocabulary in grade 3, whereas the performance of the early-exposure group in working memory is superior to that of the late-exposure group; (2) according to Figure 1, two major components, i.e., morphological awareness and word recognition in Chinese on working memory, are associated with the degree of exposure. Morphological awareness in Chinese was a significant predictor of working memory. Word recognition in Chinese had a significant effect on attention. The general patterns of the contributions of linguistic skills to cognitive abilities were stable, regardless of the degree of L2 exposure.

## Discussion

The current study is one of the first attempts to examine whether early exposure to Chinese–English biliteracy enhances cognitive skills. The main objectives of this study were to: (1) explore whether there are differences in linguistic and cognitive skills between children with early vs. late exposure to English (L2) and (2) to examine whether exposure to linguistic skills in two languages enhances cognitive abilities, and to what extent this pattern changes developmentally. In what follows we discuss the major findings concerning the potential impact of early exposure to biliteracy on cognitive skills, and the relative contribution of linguistic skills to cognitive performance.

### Early Exposure to Biliteracy Enhances Cognitive Skills

The first major finding of the present study was that early exposure to Chinese–English biliteracy enhances cognitive skills, but temporarily delays the development of native Chinese language (L1) skills. In comparison with children who only began their exposure to English in grade 3, it appears that early exposure to biliteracy results in a slight delay in L1 development. Specifically, performance on Chinese morphological awareness, phonological awareness, and vocabulary were slightly delayed among Chinese L1 children who were exposed at school to oral and reading skills in English from age 3 in comparison with their peers who were only exposed to English in grade 3. However, this L1 delay did not persist and in fact disappeared by grade 6. As age and years of language learning experience increased, the early-exposure group caught up to the late-exposure group on Chinese skills. Children with early concurrent exposure to Chinese and English language and literacy skills not only caught up to their late-exposure peers, but also maintained their L2 vocabulary advantage. Importantly, they also demonstrated an advantage on working memory, a cognitive skill.

Interestingly, better performance in working memory by the early-exposure group was only noticeable in grade 6. It has been argued that early exposure to a second language creates a cognitive burden for young children (Genesee, 2009). This result echoes previous studies that found that developing an L2 early affects the native language (L1) because the processing system of a multilingual individual is non-selective between two languages (van Hell and Dijkstra, 2002; Dussias, 2003). The two languages are thus in “competition” for cognitive and linguistic resources (Hester et al., 2004). Due to children’s limited cognitive capacity, their Chinese L1 skills, including phonological awareness, morphological awareness, and vocabulary demonstrated slightly delayed development as they were exposed simultaneously to Chinese and English, two languages with typological differences. Hence, limited cognitive capacity may temporarily hinder children’s performance in L1 acquisition (Cummins, 1991).

While early exposure may have caused temporary delays in L1 acquisition relative to those in the late-exposure group, this early exposure appears to have facilitated the development of strong working memory skills by grade 3. By grade 3, there was a significant gap in the working memory mean scores between the early-exposure group (4.4 out of 10) and the late-exposure group (3.1 out of 10); furthermore, by grade 6, the gap between the early-exposure group (8.1 out of 10) and late-exposure group (6.6 out of 10) widened. Through years of biliteracy exposure, the Matthew effect of “the rich getting richer” (Stanovich and Cunningham, 1992) appeared to benefit the early-exposure group as they developed stronger cognitive skills and improved these skills more quickly than did the late-exposure group. This finding is in line with the notion that bilinguals gradually reduce switching costs between languages, suggesting that long-term bilingual experience might contribute to efficiency in working memory capacity (Prior and MacWhinney, 2010).

This finding makes an important contribution to the literature on the theory of bilingual advantage. First, consistent with previous findings, bilingual literacy presented a benefit for children’s cognitive development (Bialystok, 2017). More importantly, this study is the first to show that the effect of morphological awareness and word recognition in Chinese on working memory varied as a function of the degree of exposure to English as an L2. Early exposure to English as an L2 may, in the short run, negatively affect students’ morphological awareness and word recognition in Chinese (their L1). The reason for this delay may lie in scriptal and morphological differences between English and Chinese as well as “time on task” or extent of exposure to the L1. This script by extent of exposure interaction in grade 3 is evident in its impact on morphological awareness and word recognition in Chinese. Moreover, the sensitivity to the orthographic form of the native language is association general cognitive function. This is due to the fact that those students who were initially skilled at word recognition and morphological awareness in their L1 may use these skills to become outstanding performers in cognitive tasks (Shu et al., 2006). Above all, these results emphasize the importance of early exposure to L2 and its benefits in later cognition development, especially working memory and language development.

### The Relative Contribution of Linguistic Skills to Cognitive Performance

Results pertaining to question 2 indicate that in grade 3, all three factors of language, reading, and cognitive–linguistic skills contribute to working memory. In grade 6, reading abilities and cognitive–linguistic skills show a significant influence on working memory, and all these effects are stable regardless of the exposure factor. Finally, for grades 3 and 6, only linguistic factors appear to significantly influence attention control abilities, indicating that stronger receptive vocabulary can facilitate the inhibition of competitive words in another language, thus facilitating better attentional control. These linguistic variables appear to consistently contribute to the development of cognition (working memory and attentional control).

These latter results can be explained by the fact that bilingualism might reinforce children’s sensitivity to cognitive skills (e.g., Leong et al., 2005). For Chinese–English emerging bilingual children, morphological awareness can predict reading and vocabulary in English and Chinese, though it may not be easy to transfer these skills from one language to the other (Cheung et al., 2010). Morphological awareness might be more specialized and complex in one language than in another (Tong and McBride-Chang, 2010). It appears that a combination of sustained exposure to two very different scripts such as Chinese and English, as well as to the different role that morphology plays in reading in these two languages, results in different working memory processes. Therefore, the relative contribution of morphological awareness and word recognition might predict cognitive skills differently in different grade (age) groups, as the results show different roles played by these three linguistic factors in developing working memory. As children gradually enhance and improve their morphological awareness as well as their vocabulary size in Chinese and English, they cultivate different working memory processes for these two languages with radically different written and spoken systems.

Further, the cognitive developmental mechanism appears to interact with both the scriptal differences between two languages and the degree of L2 exposure among emergent bilinguals. For emerging Chinese–English bilinguals, this assumed cognitive developmental mechanism involves participants’ overall literacy skills represented by language proficiency and cognitive–linguistic awareness, and cognitive functions captured by working memory and attention. In this way, the bilingual advantage becomes discernable. Bilinguals with high proficiency in two languages perform significantly better on cognitive tasks requiring strong working memory than do bilinguals with high proficiency in only one language (Ricciardelli, 1992). The reason may be that cognitive abilities develop with age (Haworth et al., 2010) and early exposure to an L2 forces bilinguals to strengthen their working memory capacity in order to deal with two different language systems and to store and process different linguistic information. In addition, their better cognitive performance also derives from stronger abilities to control their attention and inhibit competition from other linguistic sources, especially words and expressions from L1. In the present study, the emerging Chinese–English bilinguals’ language, reading, and cognitive skills continued to develop and adjust to the range and diversity of scriptal and linguistic demands, which are reflected in the gradual changes in the strength of the relationships between linguistic and cognitive skills in grades 3 and 6.

The cumulative effects of dual language and dual script exposure suggest two subsequent findings in the relative contribution of linguistic to cognitive skill performance. First, morphological awareness in Chinese was a significant predictor of working memory in the lower grade (e.g., grade 3). This is in line with the finding that morphological awareness facilitates efficiency in working memory (Zhang et al., 2014). The relationship between morphological awareness and working memory may be stronger in Chinese than in English for several reasons. First, Chinese has a clear word structure that provides important clues for readers to recognize new words in a string of characters. Second, more than 70% of Chinese characters are compound words, and learning them requires strong working memory. Third, there is a large group of homophones in Chinese that rely on visual and semantic memory to disentangle the intended meaning. Therefore, we would speculate that the early years of intensive instruction in Chinese as an L1 in both groups might play a dominant role in children’s cognitive performance. This is consistent with previous neuroimaging studies in which the Chinese L1 dominated the neural cognitive functions of Chinese–English bilinguals at the early stage of English L2 learning (Yan et al., 2016).

Secondly, when the cumulative exposure to the two languages increased, morphological awareness and word recognition in English became significant predictors of working memory in grade 6. It should be acknowledged that not all studies support the argument that working memory plays a significant role in enhancing efficient word reading fluency (Akamatsu, 2008). The present results suggest that one should jointly consider established linguistic universalities combined with specific biliteracy/bi-scriptal effects and that the cumulative effects of dual language exposure might be particularly effective in improving children’s cognitive performance.

In addition, the linguistic features and the learners’ characteristics should be considered jointly as suggested by previous studies on word recognition in both Chinese and English (Guan and Fraundorf, 2020a; Guan et al., 2020b). Our findings support Guan et al. (2020b), who through longitudinal comparison showed that word-recognition abilities are script-specific at first. That is, early script effects are influenced by an item-by-subject interaction in each language, with item-level features of the newly learned language interacting with characteristics of individual participants. Previous studies have shown that the consistency of words, orthographic awareness, morphological awareness, and linguistic abilities interact (Guan and Fraundorf, 2020a). In addition, effects at the item level generally decrease with age, as individual differences in participants’ linguistic awareness (e.g., orthographic awareness and morphological awareness) contribute more to successful performance. In Chinese, the more skilled a reader is, the weaker the item-level by participant-level interaction effect is Guan and Fraundorf (2020a), Wang et al. (2021). Although there are no specific item-level factors involved in the current study, our results suggest that such script-specific features might be physically active, though the effect of these features gradually decreases with the degree of language acquisition (Mishra and Singh, 2014).

### Limitations and Future Research

Several limitations of this study must be acknowledged. First, the present study is cross-sectional in design. Longitudinal studies are needed to replicate our findings as the developmental trajectory of children changes as they grow and a longitudinal study will have the advantage in clearly exploring the interrelationship between language abilities and cognition development. Second, our definitions of early- and late-exposure groups refer to exposure to written English in the school setting; an examination of bilingual exposure to spoken English in various contexts might yield different results. Studies directly comparing the effects of oral vs. written exposure on the relationship between reading and cognitive–linguistic skills are needed. Third, language and reading development are highly complex, encompassing many variables and relationships. The current study only touched on a small subset of these components. Future studies should explore the long-term effects of other language-related components on other cognitive performance to further clarify scriptal effects in Chinese–English bilingual research. Additional research is needed to examine the script by extent and age of exposure interaction in greater depth. For instance, researchers could investigate bilinguals exposed to other languages to see whether these findings persist.

## Conclusion

We measured 10 variables to examine the differences in linguistic abilities, reading skills, cognitive linguistic abilities, and cognition performance (working memory and attention) between two L2 exposure groups. General findings suggest that underlying mechanisms in Chinese–English specific cognitive skills and morphological awareness predict performance on working memory and attention. The general pattern of three linguistic abilities’ contributions to cognitive skills suggests that in grade 3, Chinese morphological awareness and word recognition predict cognitive performance in working memory and attention. However, among older 6th graders, English vocabulary predicts working memory. On the whole, these results suggest that sustained exposure to Chinese–English biliteracy enhances cognitive skills. It indicates that language abilities, reading skills, and cognitive–linguistic abilities play an important role in the development of cognition for children, and it further proves that language learning and cognition are mutually beneficial to each other. Improving one’s cognitive abilities can facilitate language learning, while learning a second language also benefits cognition development.

## Data Availability Statement

The raw data supporting the conclusions of this article will be made available by the authors, without undue reservation.

## Ethics Statement

The studies involving human participants were reviewed and approved by Beijing Language & Culture University. The patients/participants provided their written informed consent to participate in this study.

## Author Contributions

JY, CQG, and EG conducted the study, CQG wrote the paper, ERS edited the paper, CQG and WJM revised the paper. All authors contributed to the article and approved the submitted version.

## Funding

This work was supported by the National Social Science Foundation of China [grant number 19AYY010], and the National Natural Science Foundation of China [grant number 62077011] awarded to the 2nd author, CQG.

## Conflict of Interest

The authors declare that the research was conducted in the absence of any commercial or financial relationships that could be construed as a potential conflict of interest.

## Publisher’s Note

All claims expressed in this article are solely those of the authors and do not necessarily represent those of their affiliated organizations, or those of the publisher, the editors and the reviewers. Any product that may be evaluated in this article, or claim that may be made by its manufacturer, is not guaranteed or endorsed by the publisher.

## References

[ref1] Abu-Shnein A. (2021). Bilingualism impact on intelligence and scholastic achievement. J. Philos. Linguist. Soc. Sci. 1, 1156–1163.

[ref2] Akamatsu N. (2008). The effects of training on automatization of word recognition in English as a foreign language. Appl. Psycholinguist. 29, 175–193. doi: 10.1017/S0142716408080089

[ref3] Asher J. J. (1966). The learning strategy of the total physical response: a review. Mod. Lang. J. 50, 79–84. doi: 10.2307/323182, PMID: 21643900

[ref4] Backer K. C. Bortfeld H. (2021). Characterizing bilingual effects on cognition: the search for meaningful individual differences. Brain Sci. 11:81. doi: 10.3390/brainsci11010081, PMID: 33435472 PMC7827854

[ref5] Baddeley A. (1992). Working memory: the interface between memory and cognition. J. Cogn. Neurosci. 4, 281–288. doi: 10.1162/jocn.1992.4.3.281, PMID: 23964884

[ref6] Barac R. Bialystok E. Castro D. C. Sanchez M. (2014). The cognitive development of young dual language learners: a critical review. Early Child. Res. Q. 29, 699–714. doi: 10.1016/j.ecresq.2014.02.003, PMID: 25284958 PMC4180217

[ref7] Bialystok E. (2017). The bilingual adaptation: how minds accommodate experience. Psychol. Bull. 143:233. doi: 10.1037/bul0000099, PMID: 28230411 PMC5324728

[ref8] Bialystok E. Barac R. (2012). Emerging bilingualism: dissociating advantages for metalinguistic awareness and executive control. Cognition 122, 67–73. doi: 10.1016/j.cognition.2011.08.003, PMID: 21906732 PMC3215830

[ref9] Bialystok E. Craik F. I. Grady C. Chau W. Ishii R. Gunji A.. (2005). Effect of bilingualism on cognitive control in the Simon task: evidence from MEG. NeuroImage 24, 40–49. doi: 10.1016/j.neuroimage.2004.09.044, PMID: 15588595

[ref10] Bialystok E. Craik F. I. Klein R. Viswanathan M. (2004). Bilingualism, aging, and cognitive control: evidence from the Simon task. Psychol. Aging 19:290. doi: 10.1037/0882-7974.19.2.290, PMID: 15222822

[ref12] Bialystok E. Craik F. I. J. Luk G. (2012). Bilingualism: consequences for mind and brain. Trends Cogn. Sci. 16, 240–250. doi: 10.1016/j.tics.2012.03.001, PMID: 22464592 PMC3322418

[ref14] Bialystok E. Hakuta K. (1999). Confounded age: linguistic and cognitive factors in age differences for second language acquisition. Sec. Lang. Acquis. Crit. Period Hyp., 161–181.

[ref15] Bialystok E. Martin M. M. (2004). Attention and inhibition in bilingual children: evidence from the dimensional change card sort task. Dev. Sci. 7, 325–339. doi: 10.1111/j.1467-7687.2004.00351.x, PMID: 15595373

[ref17] Bialystok E. Ryan E. B. (1985). Toward a definition of metalinguistic skill. Merrill-Palmer Q., 229–251.

[ref18] Blom E. Küntay A. C. Messer M. Verhagen J. Leseman P. (2014). The benefits of being bilingual: working memory in bilingual Turkish–Dutch children. J. Exp. Child Psychol. 128, 105–119. doi: 10.1016/j.jecp.2014.06.007, PMID: 25160938

[ref19] Brito N. Barr R. (2012). Influence of bilingualism on memory generalization during infancy. Dev. Sci. 15, 812–816. doi: 10.1111/j.1467-7687.2012.1184.x, PMID: 23106735

[ref20] Carlisle J. F. Feldman L. B. (1995). Morphological awareness and early reading achievement. *Morphological aspects of language processing*, 189209.

[ref21] Casaponsa A. Carreiras M. Duñabeitia J. A. (2014). Discriminating languages in bilingual contexts: the impact of orthographic markedness. Front. Psychol. 5:424. doi: 10.3389/fpsyg.2014.00424, PMID: 24860536 PMC4026679

[ref22] Chen Y. J. (2015). A Study on English Teaching and Learning Approaches in China From Indigenous Aspect. Changsha: Hunan Normal University.

[ref23] Chen E. Schwartz L. (2018). “A morphological analyzer for St. Lawrence Island/Central Siberian Yupik.” in *Proceedings of the 11th Language Resources and Evaluation Conference*, Miyazaki, Japan, May.

[ref24] Chen X. Xu F. Nguyen T. K. Hong G. Wang Y. (2010). Effects of cross-language transfer on first-language phonological awareness and literacy skills in Chinese children receiving English instruction. J. Educ. Psychol. 102, 712–728. doi: 10.1037/a0018802

[ref25] Cheng C. Wang M. Perfetti C. A. (2011). Acquisition of compound words in Chinese-English bilingual children: decomposition and cross-language activation. Appl. Psycholinguist. 32, 583–600. doi: 10.1017/S0142716411000221

[ref26] Cheung H. Chung K. K. H. Wong S. W. L. McBride-Chang C. Penney T. B. Ho C. S. H. (2010). Speech perception, metalinguistic awareness, reading, and vocabulary in Chinese–English bilingual children. J. Educ. Psychol. 102, 367–380. doi: 10.1037/a0017850

[ref27] Chinese Ministry of Education (2017). English Curriculum Standards for Compulsory Education. Beijing: Beijing Normal University Press.

[ref28] Cummins J. (1991). Interdependence of first-and second-language proficiency in bilingual children. Lang. Proc. Biling. Child., 70–89. doi: 10.1017/CBO9780511620652.006

[ref29] Degani T. Prior A. Hajajra W. (2018). Cross-language semantic influences in different script bilinguals. Bilingualism 21, 782–804. doi: 10.1017/S1366728917000311

[ref30] Diependaele K. Lemhöfer K. Brysbaert M. (2013). The word frequency effect in first-and second-language word recognition: a lexical entrenchment account. Q. J. Exp. Psychol. 66, 843–863. doi: 10.1080/17470218.2012.720994, PMID: 23025801

[ref31] Dunn L. Dunn D. (2007). Peabody Picture Vocabulary Test–Fourth Edition Pearson. Circle Pines, MN: American Guidance Service.

[ref32] Dussias P. E. (2003). Syntactic ambiguity resolution in L2 learners: some effects of bilinguality on L1 and L2 processing strategies. Stud. Second. Lang. Acquis. 25, 529–557. doi: 10.1017/S0272263103000238

[ref33] Emmorey K. Borinstein H. B. Thompson R. Gollan T. H. (2008a). Bimodal bilingualism. Bilingualism 11, 43–61. doi: 10.1017/S1366728907003203, PMID: 19079743 PMC2600850

[ref34] Emmorey K. Luk G. Pyers J. E. Bialystok E. (2008b). The source of enhanced cognitive control in bilinguals: evidence from bimodal bilinguals. Psychol. Sci. 19, 1201–1206. doi: 10.1111/j.1467-9280.2008.02224.x, PMID: 19121123 PMC2677184

[ref35] Fung W. K. Chung K. K. H. Lam C. B. (2020). Mathematics, executive functioning, and visual–spatial skills in Chinese kindergarten children: examining the bidirectionality. J. Exp. Child Psychol. 199:104923. doi: 10.1016/j.jecp.2020.104923, PMID: 32693935

[ref36] Gao X. Zhou C. Chao F. Yang L. Lin C. M. Xu T.. (2019). A data-driven robotic Chinese calligraphy system using convolutional auto-encoder and differential evolution. Knowl.-Based Syst. 182:104802. doi: 10.1016/j.knosys.2019.06.010

[ref37] Genesee F. (2009). Early childhood bilingualism: perils and possibilities. J. Appl. Res. Learn. 2:104802. doi: 10.1037/a0038599

[ref38] Goswami U. Wang H. L. S. Cruz A. Fosker T. Mead N. Huss M. (2011). Language-universal sensory deficits in developmental dyslexia: English, Spanish, and Chinese. J. Cogn. Neurosci. 23, 325–337. doi: 10.1162/jocn.2010.21453, PMID: 20146613

[ref39] Guan C. Q. Fraundorf S. H. (2020a). Cross-linguistic word recognition development among Chinese children: a multilevel linear mixed-effects modeling approach. Front. Psychol. 11:544. doi: 10.3389/fpsyg.2020.00544, PMID: 32373000 PMC7176983

[ref40] Guan C. Q. Fraundorf S. H. Perfetti C. A. (2020b). Character and child factors contribute to character recognition development among good and poor Chinese readers from grade 1 to 6. Ann. Dyslexia 70, 220–242. doi: 10.1007/s11881-020-00191-0, PMID: 32100257

[ref41] Guan C. Q. Perfetti C. A. Meng W. (2015). Writing quality predicts Chinese learning. Read. Writ. 28, 763–795. doi: 10.1007/s11145-015-9549-0

[ref42] Guan Q. Yao R. Wagner R. Meng W. (2018). Developmental report of educational guidelines for 0-3yr old child learning and development. Chin. Spec. Educ. 3, 2–9.

[ref43] Guan C. Q. Ye F. Wagner R. K. Leong C. K. Meng W. J. (2014). Reading mediates the roles of morphological awareness, syntactic processing, and working memory in accounting for individual differences in Chinese written composition. J. Educ. Psychol. 106, 779–798. doi: 10.1037/a0035984, PMID: 25530630 PMC4267114

[ref44] Guan C. Q. Zhao J. Kwok R. K. W. Wang Y. (2019). How does morphosyntactic skill contribute to different genres of Chinese writing from grades 3 to 6? J. Res. Read. 42, 239–267. doi: 10.1111/1467-9817.12239

[ref45] Gunnerud H. L. Ten Braak D. Reikerås E. K. L. Donolato E. Melby-Lervåg M. (2020). Is bilingualism related to a cognitive advantage in children? A systematic review and meta-analysis. Psychol. Bull. 146:1059. doi: 10.1037/bul0000301, PMID: 32914991

[ref46] Haman E. Wodniecka Z. Marecka M. Szewczyk J. Białecka-Pikul M. Otwinowska A.. (2017). How does L1 and L2 exposure impact L1 performance in bilingual children? Evidence from polish-English migrants to the United Kingdom. Front. Psychol. 8:1444. doi: 10.3389/fpsyg.2017.01444, PMID: 28928681 PMC5591580

[ref47] Handel Z. (2013). Can a logographic script be simplified? Lessons from the 20th century Chinese writing reform informed by recent psycholinguistic research. Scr. Theol. 5, 21–66.

[ref48] Handel Z. (2014). Logography and the classification of writing systems: a response to Unger. Proc. SCRIPTA, 10–13.

[ref49] Haworth C. M. Wright M. J. Luciano M. Martin N. G. de Geus E. J. van Beijsterveldt C. E.. (2010). The heritability of general cognitive ability increases linearly from childhood to young adulthood. Mol. Psychiatry 15, 1112–1120. doi: 10.1038/mp.2009.55, PMID: 19488046 PMC2889158

[ref50] Hester R. L. Kinsella G. J. Ong B. E. N. (2004). Effect of age on forward and backward span tasks. J. Int. Neuropsychol. Soc. 10:475. doi: 10.1017/S1355617704104037, PMID: 15327726

[ref51] Johnson M. D. (2017). Cognitive task complexity and L2 written syntactic complexity, accuracy, lexical complexity, and fluency: a research synthesis and meta-analysis. J. Second. Lang. Writ. 37, 13–38. doi: 10.1016/j.jslw.2017.06.001

[ref52] Kalia V. Wilbourn M. P. Ghio K. (2014). Better early or late? Examining the influence of age of exposure and language proficiency on executive function in early and late bilinguals. J. Cogn. Psychol. 26, 699–713. doi: 10.1080/20445911.2014.956748, PMID: 35385517

[ref53] Kang L. (1993). Politics, critical paradigms: reflections on modern Chinese literature studies. Modern China 19, 13–40. doi: 10.1177/009770049301900103

[ref54] Koda K. (2016). Development of word recognition in a second language. Read. Sec. Lang. 1, 70–98. doi: 10.1017/S0142716400010602

[ref55] Laures-Gore J. Marshall R. S. Verner E. (2011). Performance of individuals with left hemisphere stroke and aphasia and individuals with right brain damage on forward and backward digit span tasks. Aphasiology 25, 43–56. doi: 10.1080/02687031003714426, PMID: 21572584 PMC3090622

[ref56] Leong C. K. Cheng P. W. Tan L. H. (2005). The role of sensitivity to rhymes, phonemes and tones in reading English and Chinese pseudowords. Read. Writ. 18, 1–26. doi: 10.1007/s11145-004-3357-2

[ref57] Liu Y. Peng D. L. (1997). Meaning access of Chinese compounds. Cogn. Process. Chin. Relat. Asian Lang. 219.

[ref58] Luo Y. C. Chen X. Geva E. (2014). Concurrent and longitudinal cross-linguistic transfer of phonological awareness and morphological awareness in Chinese-English bilingual children. Writ. Lang. Lit. 17, 89–115. doi: 10.1075/wll.17.1.05luo

[ref59] Marian V. Spivey M. (2003). Competing activation in bilingual language processing: within-and between-language competition. Bilingualism 6, 97–115. doi: 10.1017/S1366728903001068

[ref60] McArdle J. J. (1994). Structural factor analysis experiments with incomplete data. Multivar. Behav. Res. 29, 409–454. doi: 10.1207/s15327906mbr2904_5, PMID: 26745236

[ref61] Mezzacappa E. (2004). Alerting, orienting, and executive attention: developmental properties and sociodemographic correlates in an epidemiological sample of young, urban children. Child Dev. 75, 1373–1386. doi: 10.1111/j.1467-8624.2004.00746.x, PMID: 15369520

[ref62] Ministry of Education (2017). ‘Statistics on students English studying in China in 2016’. Online at (Accessed November 12, 2020).

[ref63] Mishra R. K. Singh N. (2014). Language non-selective activation of orthography during spoken word processing in Hindi–English sequential bilinguals: an eye tracking visual world study. Read. Writ. 27, 129–151. doi: 10.1007/s11145-013-9436-5

[ref64] NIES (2012). Initial success in assessment and intervention on learning disabilities at GaoQiao Primary school in Ningbo. Available at: http://jks.nbedu.net.cn/list.asp?ClassID=1&NClassID=1 (Accessed April 1, 2020).

[ref65] Peal E. Lambert W. E. (1962). The relation of bilingualism to intelligence. Psychol. Monogr. Gen. Appl. 76, 1–23. doi: 10.1037/h0093840, PMID: 35083413

[ref68] Pigott T. D. (2001). A review of methods for missing data. Educ. Res. Eval. 7, 353–383. doi: 10.1076/edre.7.4.353.8937

[ref01] Prior A. MacWhinney B. (2009). A bilingual advantage in task switching. Bilingualism: Language and Cognition 13, 253–262. doi: 10.1017/S1366728909990526, PMID: 36479004 PMC9724810

[ref69] Prior A. MacWhinney B. (2010). A bilingual advantage in task switching. Bilingualism 13:253. doi: 10.1017/S1366728909990526, PMID: 36479004 PMC9724810

[ref70] Ratcliff R. (1993). Methods for dealing with reaction time outliers. Psychol. Bull. 114:510. doi: 10.1037/0033-2909.114.3.510, PMID: 8272468

[ref71] Ricciardelli L. A. (1992). Bilingualism and cognitive development in relation to threshold theory. J. Psycholinguist. Res. 21, 301–316. doi: 10.1007/BF01067515, PMID: 1403995

[ref72] Rogers H. (2005). Writing Systems: A Linguistic Approach. Vol. 8, Malden, MA: Blackwell Publishing.

[ref73] Sebastián-Gallés N. Echeverría S. Bosch L. (2005). The influence of initial exposure on lexical representation: comparing early and simultaneous bilinguals. J. Mem. Lang. 52, 240–255. doi: 10.1016/j.jml.2004.11.001, PMID: 17919078

[ref74] Shu H. Anderson R. C. (1999). Learning to read Chinese: the development of metalinguistic awareness. *Reading Chinese Script: A Cognitive Analysis*, 1–18.

[ref75] Shu H. McBride-Chang C. Wu S. Liu H. (2006). Understanding Chinese developmental dyslexia: morphological awareness as a core cognitive construct. J. Educ. Psychol. 98, 122–133. doi: 10.1037/0022-0663.98.1.122

[ref76] Singson M. Mahony D. Mann V. (2000). The relation between reading ability and morphological skills: evidence from derivational suffixes. Read. Writ. 12, 219–252. doi: 10.1023/A:1008196330239

[ref77] Stanovich K. E. Cunningham A. E. (1992). Studying the consequences of literacy within a literate society: the cognitive correlates of print exposure. Mem. Cogn. 20, 51–68. doi: 10.3758/BF03208254, PMID: 1549065

[ref78] Taylor I. Taylor M. M. (1995). Writing and Literacy in Chinese, Korean and Japanese. Vol. 3. Amsterdam: John Benjamins Publishing Company.

[ref79] Tong X. McBride-Chang C. (2010). Chinese-English biscriptal reading: cognitive component skills across orthographies. Read. Writ. 23, 293–310. doi: 10.1007/s11145-009-9211-9

[ref80] Torgesen J. K. Wagner R. Rashotte C. (2012). Test of Word Reading Efficiency: (TOWRE-2), Austin, TX: Pro-Ed Pearson Clinical Assessment.

[ref81] Treiman R. Zukowski A. (1991). “Levels of phonological awareness,” in Phonological processes in literacy: A tribute to Isabelle Y. Liberman, eds. Brady S. A. Shankweiler D. P. Hillsdale, NJ: Erlbaum. 67–83.

[ref82] Třísková H. (2011). The structure of the mandarin syllable: why, when and how to teach it. Arch. Orient. 79, 99–134.

[ref83] van Hell J. G. Dijkstra T. (2002). Foreign language knowledge can influence native language performance in exclusively native contexts. Psychon. Bull. Rev. 9, 780–789. doi: 10.3758/BF03196335, PMID: 12613683

[ref84] Wagner R. K. Torgesen J. K. (1987). The nature of phonological processing and its causal role in the acquisition of reading skills. Psychol. Bull. 101:192. doi: 10.1037/0033-2909.101.2.192, PMID: 22209402

[ref85] Wang M. Perfetti C. A. Liu Y. (2005). Chinese–English biliteracy acquisition: cross-language and writing system transfer. Cognition 97, 67–88. doi: 10.1016/j.cognition.2004.10.001, PMID: 16139587

[ref86] Wang L. Wang J. Liu D. Lin D. (2021). The role of metalinguistic awareness and character properties in early Chinese reading. J. Exp. Child Psychol. 210:105185. doi: 10.1016/j.jecp.2021.105185, PMID: 34087684

[ref87] Yan H. Zhang Y. M. Xu M. Chen H. Y. Wang Y. H. (2016). What to do if we have nothing to rely on: late bilinguals process L1 grammatical features like L1 natives. J. Neurolinguistics 40, 1–14. doi: 10.1016/j.jneuroling.2016.04.002

[ref88] Zhang J. Lin T. J. Wei J. Anderson R. C. (2014). “Morphological awareness and learning to read Chinese and English,” in Reading Development and Difficulties in Monolingual and Bilingual Chinese Children eds. Chen X. Wang Q. Luo Y. C. (Dordrecht: Springer), 3–22.

[ref89] Zhou E. L. (1958). Current tasks for writing system reform. Report for the Chinese People’s Political Consultative Conference, Jan 10, 1958. Available at: http://cpc.people.com.cn/GB/69112/75843/75874/75994/5182927.html (Accessed February 18, 2018).

